# Antiviral defense systems drive persistence of antimicrobial‐resistant bacteria but limit the transfer of antimicrobial resistance genes in anaerobic digestion

**DOI:** 10.1002/imt2.70145

**Published:** 2026-06-27

**Authors:** Junya Zhang, Tiedong Lu, Qihe Tang, Song‐Can Chen, Daniel Rios Garza, Bin Liu, Yunwei Cui, Yuansong Wei, Hans Hermann Richnow

**Affiliations:** ^1^ State Key Laboratory of Regional Environment and Sustainability, Research Center for Eco‐Environmental Sciences, Chinese Academy of Sciences Beijing China; ^2^ University of Chinese Academy of Sciences Beijing China; ^3^ Agricultural Resources and Environmental Research Institute, Guangxi Academy of Agricultural Sciences/Guangxi Key Laboratory of Arable Land Conservation Nanning China; ^4^ College of Life Science and Technology Guangxi University Nanning China; ^5^ State Key Laboratory of Soil Pollution Control and Safety, MOE Key Laboratory of Environment Remediation and Ecological Health, College of Environmental and Resource Sciences, Zhejiang University Hangzhou China; ^6^ Université Paris‐Saclay, INRAE, PROSE Antony France; ^7^ Key Laboratory of Environmental Biotechnology, Research Center for Eco‐Environmental Sciences Chinese Academy of Sciences Beijing China; ^8^ Atmospheric Chemistry Department (ACD) Leibniz Institute for Tropospheric Research (TROPOS) Leipzig Germany

## Abstract

Phage–host interactions critically shape environmental antimicrobial resistance (AMR). Using swine manure anaerobic digestion and multi‐omics (metagenomics, meta‐transcriptomics, and Hi‐C), we mapped the phage–bacteria arms race and its impact on AMR dynamics. We revealed that phage‐mediated lysis overwhelmingly dominates transduction, while phages rarely carry antimicrobial resistance genes (ARGs), and phage‐borne ARGs showed no expression, challenging the paradigm of phages as primary vectors of ARGs. Crucially, the intense on‐going phage–host arms race drives the widespread presence and expression of antiviral defense systems (ADSs) in antimicrobial‐resistant bacteria (ARB). These ADSs exhibit a vital ecological dual role: they protect ARBs from phage lysis promoting persistence while simultaneously suppressing horizontal gene transfer (HGT, e.g., conjugation), as validated by in vitro conjugation assays. Our findings elucidate this duality, offering a novel framework to harness phage lytic pressure and ADS‐mediated HGT suppression for environmental AMR mitigation.

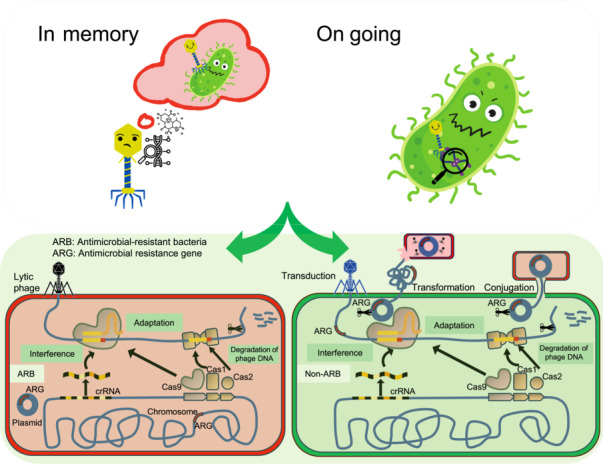

## ETHICS STATEMENT

No animals or humans were involved in this study.

To the Editor,

Antimicrobial resistance (AMR) is a formidable One Health crisis linked to 4.71 million deaths in 2021 [1] and heavily exacerbated by the livestock sector, which accounts for over 70% of global antimicrobial use [2]. Anaerobic digestion (AD) systems, processing millions of tons of antimicrobial‐laden manure annually [3], serve as both massive environmental reservoirs for AMR dissemination and controllable ecological interfaces to investigate resistance dynamics [4].

Within these dense microbial ecosystems, phages are pivotal agents shaping bacterial evolution [5, 6]. However, their precise role in AMR dynamics remains paradoxically contested. While traditionally viewed as primary vectors for horizontal gene transfer (HGT) via transduction [7, 8], recent large‐scale virome analyses suggest that phages are predominantly lytic agents (97% in oceans [GOV2 dataset], 95% in soils [IsoGenie dataset], and 87% in human gut [GVD dataset]) that rarely encode functional ARGs [9, 10], where viruses exert substantial pressure on bacterial populations. This intense lytic pressure, while driving the broad promotion of phage therapy [11], paradoxically catalyzes a continuous phage–bacteria arms race, driving the rapid evolution of diverse antiviral defense systems (ADSs), such as CRISPR‐Cas, R‐M, and abortive infection [12, 13].

This counter‐evolution introduces an unresolved ecological duality in AMR dynamics. On one hand, ADS can co‐locate with ARGs, protecting antimicrobial‐resistant bacteria (ARB) from viral lysis and ensuring their environmental persistence [14]. On the other hand, these systems may profoundly suppress HGT by promiscuously cleaving incoming foreign DNA, including plasmid‐borne ARGs [15].

**Figure 1:**
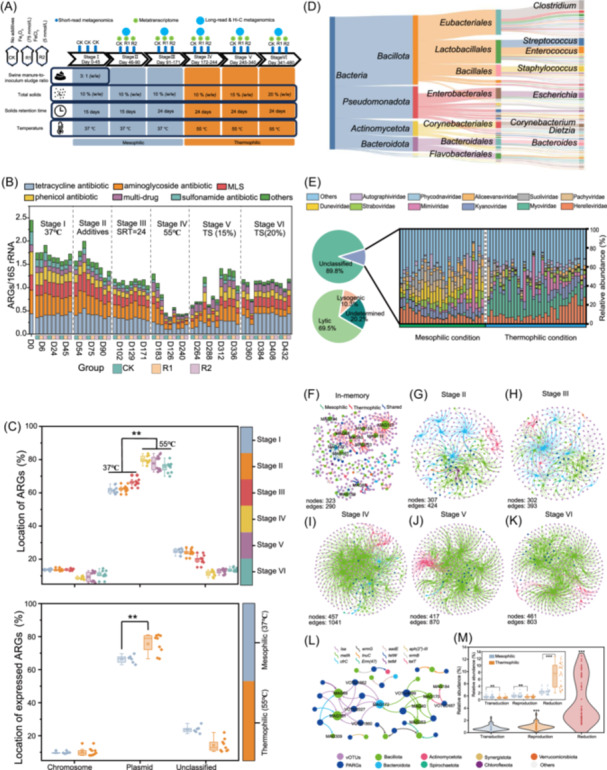
Profile and phage‐mediated antimicrobial resistance genes in anaerobic digestion. (A) Schematic demonstration showing the experimental design and sampling strategies for the short‐read, long‐read, and Hi‐C metagenomics along with meta‐transcriptomics. (B) Changes of the relative abundance of antimicrobial resistance genes (ARGs) normalized by 16S rRNA response to varied operational parameters at the antimicrobial type level. D, day; MLS, macrolide‐lincosamide‐streptogramin; SRT, solids retention times; TS, total solids. Overall location (C) and hosts (D) of ARGs. (E) Overall view on the lifestyle and changes of the viral community at the family level. (F) In‐memory viral infection between phage and antimicrobial‐resistant bacteria (ARB) based on the sequence alignments of CRISPR‐Cas spacers, tRNA, and homology matches. Green and red edges indicated the phage–ARBs connections, which were only found for mesophilic and thermophilic conditions, respectively, while the blue edges indicated that the connections were shared. On‐going phage–host interactions determined by Hi‐C data from Stage II (G), Stage III (H), Stage IV (I), Stage V (J), and Stage VI (K). The nodes are colored through their taxonomy at the phylum level, and the node size indicates the connection degree. (L) Phage transduction network deduced from the in‐memory and on‐going viral infection based on the shared ARGs between phage and ARBs. The edge color represents the shared ARG subtypes, and the node color has the same meaning to the Hi‐C results (F–K). (M) Relative abundance of phages mediating the transduction, reproduction, and reduction of ARGs. The main violin plot illustrates the overall distribution of these three phage‐mediated roles, highlighting the dominance of reduction. The inset box plot illustrates the changes of relative abundance of phages involved in transduction, reproduction, and reduction under mesophilic and thermophilic conditions. The transition to thermophilic conditions significantly decreased the abundance of phages driving transduction and reproduction, while dramatically increasing those driving reduction. Asterisks indicate significant differences between the two temperature regimes (two‐sided Wilcoxon rank‐sum test; **p < 0.01, ***p < 0.001).

To resolve whether phage ecology and induced immunity ultimately promote or restrict the spread of ARGs, we investigated an over 440‐day semi‐continuous swine manure AD, which was subjected to sequential operational perturbations across six stages, including the introduction of iron‐based additives, transition from mesophilic (37°C) to thermophilic (55°C) conditions, and incremental increases in total solids (TS) loading from 10% to 20% (Figure 1A and Supporting Information S1: Figure 1; Supporting Information S2: Table 1). System stability was determined by the achievement of steady‐state daily methane production. By integrating short‐ and long‐read metagenomics, meta‐transcriptomics, and high‐throughput chromosome conformation capture (Hi‐C) mapping alongside in vitro conjugation validations, we systematically deciphered functional consequences of phage–host arms race and dual roles of ADS in shaping environmental resistome.

## RESULTS AND DISCUSSION

### Phage‐mediated fate of ARGs: Lysis overwhelmingly outweighs transduction

**Figure 2:**
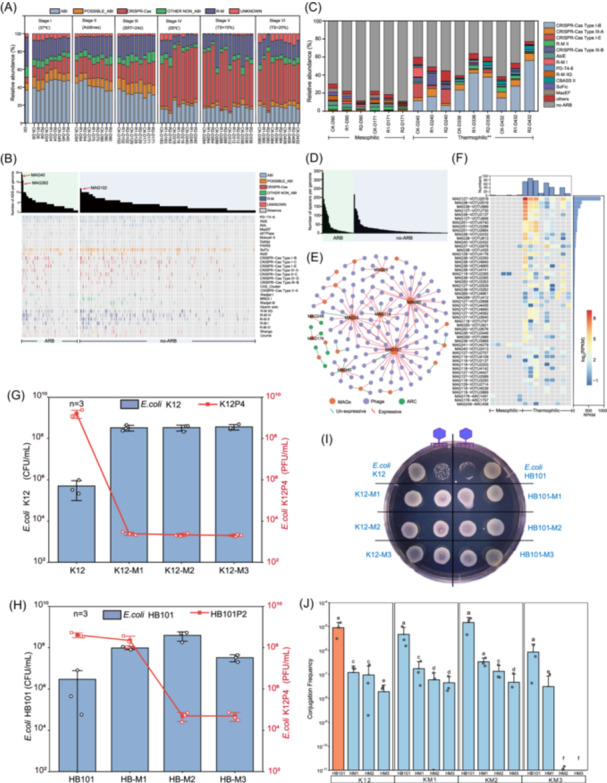
Profile of antiviral defense system (ADS) and its role in the horizontal gene transfer of antimicrobial resistance genes. (A) Compositional shifts of ADS across the six stages under varying conditions. SRT, solids retention times; TS, total solids. (B) Distribution of ADS in antimicrobial‐resistant bacteria (ARB) versus non‐ARB. Each row represents a different metagenome‐assembled genome (MAG). Each column corresponds to a different ADS (color indicates the presence of the ADS). (C) Relative abundance of the expressed ADS in ARB and non‐ARB, with non‐ARB portion indicating the ADS ratio in non‐ARB. Two asterisks denote significant differences (*p* < 0.001). (D) Distribution of CRISPR‐Cas spacer counts per genome, comparing ARB and non‐ARB, with significantly higher abundance in ARB (*p* < 0.05). (E) CRISPR‐Cas spacer‐based immune network illustrating interactions among MAGs (orange), viral operational taxonomic units (vOTUs) (purple), and ARGs‐carrying contigs (ARCs, green), clearly identifying defended vOTUs. Red edges denote expressed spacers, while green edges indicate unexpressed spacers, highlighting the active defense. (F) Expression profiles of spacers within the immune network under mesophilic (37°C) and thermophilic (55°C) conditions with log_2_ (RPKM) values indicating differential expression (red: high, blue: low). Columns represent MAG‐vOTU/ARC pairs, and rows denote individual spacers. Confirmation of the developed phage resistance of *Escherichia coli* K12 (G) and *E. coli* HB101 (H). Changes of the lytic infection activity after the development of the phage resistance for *E. coli* K12 and *E. coli* HB101 (I). (J) Conjugation frequency between *E. coli* K12 (donor) and *E. coli* HB101 (recipient) along with the ADS development. The marked superscripts with different letters mean significant differences from the other experiments (Kruskal–Wallis test, *p* < 0.05).

Our data indicated that ARG profiles underwent distinct compositional shifts and clustered correspondingly with thermal stress and TS loading (Figure 1B and Supporting Information S1: Figure 2A,B). Transitioning to thermophilic conditions markedly reduced the relative abundance of ARGs, whereas elevated TS loads enriched them possibly associated with the accumulated residual antibiotics and other co‐selecting agents from the swine manure, which was validated through qPCR (Supporting Information S1: Figure 2C). Interestingly, 70.5%–77.6% of these ARGs were located on plasmids or flanked by integrative and conjugative elements (Supporting Information S2: Table 2), underscoring a high baseline potential for conjugative mobility (Figure 1C). Meta‐transcriptomic analysis revealed that most of ARGs in AD were silent (Supporting Information S1: Figure 3), while expressed ARGs were dominated by *Pseudomonadota* (Supporting Information S1: Figure 4 and Supporting Information S2: Table 3), despite their low genomic abundance (Figure 1D, 2.16% ± 3.42%).

Concurrently, comprehensive viral community profiling via bulk metagenomics identified 6210 non‐redundant DNA viral operational taxonomic units (vOTUs, Supporting Information S2: Table 4) alongside 517 RNA vOTUs (Supporting Information S2: Table 5). The vast majority of these DNA vOTUs (69.5%) were predicted to be lytic (Figure 1E). To map these ecological pressures, we recovered 312 high‐quality metagenome‐assembled genomes (MAGs, Supporting Information S2: Table 6; 91.6% completeness and 1.6% contamination on average) and reconstructed highly resolved phage–host interaction networks. We deployed Hi‐C to chemically cross‐link and finally capture 6100 *on‐going*, active in vivo phage infections covering 253/312 recovered MAGs (Figure 1F–K). This physical evidence was further substantiated by *in‐memory* historical interactions (429/702 events) mined via CRISPR‐Cas spacers, tRNA, and sequence homology [16]. These methods effectively integrated 67 of 74 identified ARGs‐carrying MAGs into a phage‐driven ARGs network. Under mesophilic conditions, phages primarily interacted with ARBs assigned as Bacteroidota (48.4%) and Bacillota (34.3%), shifting to Bacillota (91.7%) and Actinomycetota (5.2%) under thermophilic conditions (Supporting Information S1: Figure 4B).

The phage‐driven ARGs network fundamentally challenges the conventional paradigm that phages serve as a reservoir and primary vehicles for HGT. Instead, phage‐mediated reduction overwhelmingly dominates ARGs' dynamics. In total, 26 of 6210 vOTUs carried ARGs, representing only 0.55% ± 0.32% of total ARGs and 2.56% ± 1.04% of the viral community. We did not find any RNA vOTUs carrying ARGs. Of the 2565 non‐redundant ARG‐associated infection events identified, 1524 (57.7%) were associated with lytic vOTUs. This drastically outnumbers predicted lysogenic infections (8.9%) and transduction events (Figure 1L, a mere 45 occurrences, or 0.74%). Furthermore, phages driving reduction (5.16% ± 3.54%) significantly outweighed those for transduction (0.74% ± 0.39%) and reproduction (0.99% ± 0.45%) (Wilcoxon rank‐sum test, *p* < 0.001). We also noted that the transition to thermophilic condition significantly reduced the transduction and reproduction roles but largely enhanced the reduction role of viral communities in AD (Figure 1M).

The 45 transduction events were restricted to a minor fraction of ARG subtypes, none of which exhibited active expression in our meta‐transcriptomic data. However, cross‐phyla transduction was evident, which should be concerned. For instance, MAG172 (Bacteroidota_Fermentimonas) acquired *Isa* and *mefA* from MAG161, MAG69, and MAG40 belonging to Bacillota, and *aadE* transferred between MAG217 (Bacteroidota) and MAG261 (Bacillota) via vOTU6087.

The lytic pressure phages exerted on ARBs was immense. Each ARB was targeted by an average of 35.6 vOTUs, facing a significantly higher infection burden than non‐resistant hosts (20.0 vOTUs). This suppression was highly sensitive to operational parameters; under the extreme thermal stress of 55°C, the reduction role of phages significantly increased (Figure 1M). This environmental stress catalyzed a classic kill‐the‐winner ecological dynamic, compelling phages to aggressively invade and lyse dominant ARB populations to ensure their own replication. This intensified lytic activity revealed by virus–host ratio (VHR) strongly and negatively correlated with overall ARGs abundance (Spearman, *R* = −0.546, *n* = 61, *p* < 0.001).

These massive numerical discrepancies confirm that phages function predominantly as an active biological sink, actively curbing ARB proliferation rather than serving as functional vectors for ARGs dissemination in AD. Nonetheless, we should note that phage‐mediated lysis is a significant, stress‐induced biological mechanism that operates alongside, rather than independently of, the direct physiological effects of high temperature. Besides, rare transduction events could carry disproportionate environmental and clinical risks that should be emphasized. Because even one HGT event between pathogens could cause risks that were far beyond the enhanced reduction [17, 18].

### The dual role of antiviral defense systems in the fate of ARGs

To survive the intense phage–host arms race, ARB have evolved robust and diverse ADS repertoires making them harder to clear. Our genomic profiling identified 3192 ADS across 107 distinct classes (Figure 2A), revealing that ARBs harbor a significantly larger immune arsenal (an average of 4.5 ADS per MAG) than their susceptible counterparts (Figure 2B, 3.7, Wilcoxon rank‐sum test, *p* < 0.05). Crucially, this disproportionate viral burden is not a targeted virological attack on resistance traits, but a secondary ecological consequence of the kill‐the‐winner dynamic. Primary ARBs in our system (Figure 1D) are predominantly fast‐growing r‐strategists. Fueled by high organic loads, they rapidly proliferate into dominant winners, thereby inherently attracting intense lytic phage infections aimed at maximizing viral replication.

Transitioning the system from mesophilic to thermophilic conditions induced a major compositional shift, elevating CRISPR‐Cas systems to dominance (Figure 2A, 50.6%) and surging the ADS expression within ARBs from 27.5% to 46.4% (Figure 2C). By mapping highly resolved CRISPR‐Cas spacer‐based immunity networks, we observed that heavily defended ARBs (Figure 2D, such as MAG127 (equipped with 338 distinct spacers) repelled up to 39 specific vOTUs (Figure 2E). The actively expressed spacers indicated that they could have successfully shielded ARBs from viral lysis, driving their persistence in AD (Figure 2F). Crucially, none of the historical interactions captured in this CRISPR‐Cas spacer‐based immunity network were detected as active, on‐going infections in our Hi‐C data.

However, this enhanced survival mechanism incurs a critical evolutionary trade‐off: the restriction of HGT. Because ADS function as a pan‐immune barrier, they do not exclusively target bacteriophages; they promiscuously intercept and degrade various MGEs [19], including the plasmids that harbor ~70% of the ARGs in AD. Our in situ meta‐transcriptomic data functionally validated this collateral suppression. For instance, actively expressed CRISPR‐Cas systems in specific hosts (MAG176 and MAG248) directly targeted and blocked ARC1491, ARC1457, and ARC458 carrying *ant(9)‐I* and *ErmT*, which were located on incoming ICEs and plasmids (Figure 1F). This dynamic reveals a fundamental biological bottleneck: while the arms race forces ARBs to heavily fortify their genomes against lethal viral attacks, this fortification inadvertently neutralizes their capacity to acquire novel ARGs.

To phenotypically validate this ADS‐mediated suppression of conjugation, we conducted targeted in vitro assays using environmentally prevalent model strains. We established conjugative pairs using *Escherichia coli* K12 as the ARGs‐donor (carrying the multidrug‐resistant RP4 plasmid) and *E. coli* HB101 as recipients. After inducing robust phage resistance through co‐incubation with specific lytic phages (Figure 2G–I), we evaluated their plasmid acquisition capacity. Once the *E. coli* HB101 recipient developed phage immunity, the frequency of conjugative RP4 acquisition plummeted significantly by 98.7%–99.8% compared to the wild‐type susceptible pairs, dropping from 9.05 × 10^−6^ to 1.91 × 10^−8^ (Figure 2J, Wilcoxon rank‐sum test, *p* < 0.05). We hypothesize that this broad‐spectrum phenotypic suppression is heavily driven by R‐M systems, which were highly prevalent in our AD model (identified in 216 out of 312 MAGs). In this mechanism, phage‐induced hyper‐activation of host DNA methylation allows R‐M enzymes to degrade unmethylated or mismatched incoming plasmids as foreign DNA, reinforcing the genetic blockade.

These findings elucidate a critical ecological duality governing the fate of the environmental resistome. The intense selective pressure exerted by lytic phages forces a structural divergence in bacterial evolution. On one hand, the aggressive upregulation of ADS ensures the short‐term survival and persistence of ARB populations, making them notoriously difficult to clear. On the other hand, this same immune fortification acts as a long‐term regulatory sink that fundamentally restricts the plasmid‐mediated dissemination of ARGs across the broader microbial community. Notably, this phenomenon is not confined to the unique parameters of AD systems. Genomic databases indicate that 78% of fully sequenced bacterial genomes encode multiple ADS [20], underscoring that ADS‐mediated HGT suppression is a ubiquitous trait shaping microbial ecology. By redefining this dual role, we provide a mechanistic explanation for the stalled spread of ARGs under high viral pressure, establishing a theoretical framework where the phage–bacteria arms race can be leveraged to strategically restrict HGT.

### Ecological implications and engineering strategies for AMR mitigation

Translating these ecological trade‐offs into practical applications offers two innovative AMR mitigation strategies for engineered systems. First, intentionally applying thermal stress (e.g., thermophilic AD) artificially exacerbates the kill‐the‐winner dynamic, maximizing phage‐induced ARB lysis. Second, we propose integrating a native phage suspension reflux system. Continually recycling lytic phages back into the reactor circumvents the need for screening host‐specific viruses and perpetually forces the microbial community into an active arms race. This sustained viral pressure ensures the continuous high‐level expression of ADS, harnessing their dual benefit: maximizing lysis‐driven ARB reduction while enforcing a firewall against the conjugation or transformation of ARGs.

While large‐scale implementation faces practical bottlenecks, such as unfavorable energy balances and the technical hurdles of rapid phage extraction from complex sludge matrices, this mechanistic framework provides a transformative theoretical foundation for harnessing viral ecology to control the environmental AMR dissemination. While the AD system provides a robust model for high‐pressure environmental reservoirs, we acknowledge that these specific phage–host dynamics and ADS‐mediated HGT suppression mechanisms require further validation across other diverse ecosystems, such as soil, aquatic, and human gut microbiomes, where spatial and nutritional constraints differ significantly.

Our findings challenge the paradigm of phages as primary ARG vectors, demonstrating that phage‐mediated lysis overwhelmingly outweighs transduction. The intense phage–host arms race forces ARB to overexpress ADS. We elucidate a critical ecological duality: while ADS ensure bacterial persistence against viral lysis, they indiscriminately degrade incoming mobile genetic elements, profoundly restricting HGT as validated by in vitro conjugation assays. Harnessing this duality, such as through thermal stress or engineered viral pressure, offers a transformative, ecology‐driven framework to simultaneously maximize the eradication of AMR populations and stall the environmental dissemination of ARGs.

## AUTHOR CONTRIBUTIONS

**Junya Zhang**: Conceptualization; investigation; writing—original draft; methodology; visualization; formal analysis; data curation; funding acquisition; project administration; software. **Tiedong Lu**: Investigation; writing—original draft; formal analysis; methodology; validation. **Qihe Tang**: Methodology; writing—review and editing; formal analysis. **Song‐Can Chen**: Formal analysis; writing—review and editing. **Daniel Rios Garza**: Formal analysis; writing—review and editing. **Bin Liu**: Writing—review and editing. **Yunwei Cui**: Formal analysis; writing—review and editing. **Yuansong Wei**: Conceptualization; investigation; writing—review and editing; supervision; resources; project administration. **Hans Hermann Richnow**: Conceptualization; investigation; writing—review and editing; supervision.

## CONFLICT OF INTEREST STATEMENT

The authors declare no conflicts of interest.

## Supporting information


**Figure S1:** The schematic and in‐site demonstration of the three continuous stirred tank reactors (CSTR) used in this study.
**Figure S2:** Profile of antimicrobial resistance genes (ARGs) in anaerobic digestion.
**Figure S3:** Profile of the expression of antimicrobial resistance genes (ARGs) in anaerobic digestion.
**Figure S4:** Profile of the hosts of antimicrobial resistance genes (ARGs) and phages.

**Table S1:** Experimental design and samples collection.
**Table S2:** The detail information of the ARGs‐carrying contigs (ARC) detected in this study.
**Table S3:** The detail information of the ARGs‐carrying contigs (ARC) detected at the RNA level in this study.
**Table S4:** The detail information of the identified DNA vOTUs in this study.
**Table S5:** The detail information of the identified RNA vOTUs in this study.
**Table S6:** The detail information of the MAGs binned in this study.

## Data Availability

All the sequencing data have been deposited in GSA under submission number CRA013482 (https://ngdc.cncb.ac.cn/gsa/browse/CRA013482). The code and scripts used are saved in GitHub https://github.com/zjyzjjzmt/ADS_AMR_AD. Supplementary materials (methods, figures, tables, graphical abstract, slides, videos, Chinese translated version, and updated materials) may be found in the online DOI or iMeta Science http://www.imeta.science/.
