# Supplementary material for: Antiviral defense systems drive persistence of antimicrobial‐resistant bacteria but limit the transfer of antimicrobial resistance genes in anaerobic digestion
Source: Imeta. 2026 Jun 27;5(3):e70145. doi: 10.1002/imt2.70145 (PMC13377415; doi:10.1002/imt2.70145)
Supplement: Supplementary file 1 — Figure S1: The schematic and in‐site demonstration of the three continuous stirred tank reactors (CSTR) used in this study. Figure S2: Profile of antimicrobial resistance genes (ARGs) in anaerobic digestion. Figure S3: Profile of the expression of antimicrobial resistance genes (ARGs) in anaerobic digestion. Figure S4: Profile of the hosts of antimicrobial resistance genes (ARGs) and phages. [file IMT2-5-e70145-s001.docx]

Supporting Information to

**Antiviral defense systems drive persistence of antimicrobial-resistant bacteria but limit the transfer of antimicrobial resistance genes in anaerobic digestion**

**Running title:** The dual role of antiviral defense systems on antimicrobial resistance genes

Junya Zhang^1,2^, Tiedong Lu^3^, Qihe Tang^1,4^, Song-Can Chen^5^, Daniel Rios Garza^6^, Bin Liu^2,7^, Yunwei Cui^1,4^, Yuansong Wei^1,2^*, Hans Hermann Richnow^8^*

^1^ State Key Laboratory of Regional Environment and Sustainability, Research Center for Eco-Environmental Sciences, Chinese Academy of Sciences, Beijing 100085, China

^2^ University of Chinese Academy of Sciences, Beijing 100049, China

^3^ Agricultural Resources and Environmental Research Institute, Guangxi Academy of Agricultural Sciences/Guangxi Key Laboratory of Arable Land Conservation, Nanning 530007, China

^4^ College of Life Science and Technology, Guangxi University, Nanning 530005, China

^5^ State Key Laboratory of Soil Pollution Control and Safety, MOE Key Laboratory of Environment Remediation and Ecological Health, College of Environmental and Resource Sciences, Zhejiang University, Hangzhou 310058, China

^6^ Université Paris-Saclay, INRAE, PROSE, 92761 Antony, France

^7^ Key Laboratory of Environmental Biotechnology, Research Center for Eco-Environmental Sciences, Chinese Academy of Sciences, Beijing 100085, China

^8^ Atmospheric Chemistry Department (ACD), Leibniz Institute for Tropospheric Research (TROPOS), Permoserstraße 15, 04318 Leipzig, Germany

***Correspondence:** [yswei@rcees.ac.cn](mailto:yswei@rcees.ac.cn) (Yuansong Wei); [richnow@tropos.de](mailto:richnow@tropos.de) (Hans Hermann Richnow).

## METHODS

## Experimental setup

This study utilized three continuous stirred-tank reactors with an 8.0 L working volume operated over six sequential stages for ~480 days to investigate the impacts of iron-based additives, solids retention time, temperature, and total solids load on the ARGs transfer in AD of swine manure, a known typical environmental reservoir of ARGs [1,2]. The schematic and experimental setup of the three CSTRs are illustrated in Fig. 1a and S1.

**Stage I (Start-up, 0~45d):** Reactors were initialized with a swine manure-to-inoculum sludge ratio of 3:1 (*w*/*w*), TS of 10% (*w*/*w*), a SRT of 15 days, and a mesophilic temperature of 37°C. The swine manure and inoculum sludge were collected from the same facility operating an anerobic digester treating swine manure. This stage lasted 45 days (three SRTs). Feeding occurred every three days, with 1.6 L of swine manure (TS 10%) added from the top and an equal volume discharged from the bottom. Agitation was set at 80 rpm for 30 minutes with 15-minute intervals. Methane production was monitored in real time after CO₂ removal with 3M NaOH. **Stage II (46~90d):** The three reactors were differentiated as CK (no additives), R1 (75 mmol/L Fe_2_O_3_, Aladdin, F108317, powder), and R2 (5 mmol/L FeCl_3_, Aladdin, I112065, solution). Additive concentrations were optimized based on our prior studies showing improved ARGs reduction and methane production via microbial community modulation [3,4]. Additives were continuously supplied with the substrate to maintain steady concentrations in the reactors. **Stage III (91~171d):** SRT was extended from 15 days to 24 days. **Stage IV(172~244d):** Temperature was elevated to 55°C for thermophilic conditions. **Stages V (245~340d) and VI (342~480d)**: TS was incrementally increased to 15% and 20%, respectively.

The overall information of the experimental design and samples collection was shown in Table S1. Each stage operated for at least three SRTs. Samples were collected at least once per SRT for short-read metagenomics and meta-transcriptome analyses, with composite stage samples processed for Hi-C and long-read Nanopore sequencing (Fig. 1a).

## DNA and RNA extraction

The experimental time comprises a total of 20 SRTs (6 SRTs of 15 days and 14 SRTs of 24 days) for each reactor, yielding 60 samples plus the day 0 (D0) sample for DNA extraction and short-read metagenomic sequencing. Starting from Stage II after start-up, meta-transcriptome samples (15 in total) were collected at the end of each stage where steady-state conditions were achieved better, while the five composite stage samples (mixing the samples from CK, R1 and R2 in the same stage) were used for the Hi-C and long-read Nanopore sequencing. DNA extraction was done using the FastDNA Spin kit for soil (MP Bio, USA), while RNA was extracted through the Soil RNA Kit200 (Omega BioTek, GA, USA), followed by rRNA removal with the Ribo-Zero rRNA Removal Kit (Epicentre, Madison, WI, USA). Extracted DNA and RNA was detected and quantified by electrophoresis on a 1% agarose gel and a NanoDrop 2000 (Thermo Scientific, USA), respectively.

## Short- and long-read metagenomics along with meta-transcriptome sequencing

These DNA samples were sent to Majorbio BioPharm Technology Co., Ltd. (Shanghai, China) for the library construction (350 bp) and pair-end sequencing (150 bp). The meta-transcriptome library construction (350 bp) was prepared using TruSeqTM RNA Sample Prep Kit. Both metagenomics and meta-transcriptomic sequencing were conducted through the HiSeq 4000 platform (Illumina, USA). Long-read libraries were prepared using Oxford Nanopore Technologies (ONT) Ligation library preparation kit (SQK-LSK109, EXP-NBD104, and EXP-NBD114) following manufacturer’s instructions and sequenced with the ONT PromethION sequencer using FLO-PRO002 flow cells. Approximately 1.01 Tb, 451 Gb and 63.5 Gb of raw data were generated for the short-read metagenomics, meta-transcriptome and long-read nanopore sequencing, respectively.

## Profiling of ARGs at reads level

Short-read metagenomics and meta-transcriptomics were both trimmed and quality controlled through metaWRAP-Read_qc module [5]. ARGs-like reads were determined by Diamond (v2.0.14, –query-cover 75, –id 90, –e-value 1e-5) against Comprehensive Antibiotic Resistance Database (CARD, v3.1.4) [6,7]. To reduce the bias caused by the sequencing depth and biomass, the expression of ARGs at RNA level was normalized by reference length and sequencing depth as RPKM (reads per kilo per million reads), while the relative abundance of ARGs at DNA level was further normalized by RPKM of 16S rRNA.

**Conventional quantitative PCR**

To elucidate the results from the metagenomics, we quantified the absolute gene copies of *ermB* and *tetM* along with 16S rRNA through the qPCR. Each gene was quantified in triplicate for each sample using a standard curve and a negative control. Briefly, plasmids containing these specific genes were used as standards in a 10-fold dilution and added to every qPCR run. The 25 µL PCR reaction mixtures contained 12.5 µL of 2×SYBR Green pro TaqHS Premix with ROX (Accurate Biology, China), 0.5 µL each of 10 mM forward and reverse primers, 10.5 µL of DNA-free water, and 1.0 mL of standard plasmid or DNA extract. The qPCR amplification was performed as follows: (1) 50 ºC, 2 min; (2) 95 ºC, 5 min; (3) 95 ºC, 20 s; (4) annealing temperature, 30 s; (5) 72 ºC, 31 s; (6) plate read, repeat steps (3) through (5) 39 more times; (7) melt-curve analysis: 60 ºC-95 ºC, 0.2 ºC read. The reaction was conducted using an ABI Real-time PCR system 7900HT (ABI, USA). The primers and annealing temperature were shown in our previous study [8,9]. The corresponding amplification efficiencies for *ermB*, *tetM* and 16s rRNA were 95%, 105% and 106%, respectively.

## Potential conjugative mobility of ARGs determined at contigs level

Short-read metagenomics and meta-transcriptome were further assembled using MEGAHIT (v1.1.3) individually, and only contigs >1 kb were kept for further analysis [10]. The ARGs-carrying contigs (ARCs) were searched against CARD (–query-cover 70, –id 80, –e-value 1e-10), and then further blasted against ICEberg 3.0 (integrative & conjugative elements) database [11]. PlasFlow (v1.1) was adopted to determine the ARCs belonging to plasmids with a recall rate of 85.98% and precision of 72.17% [12]. We considered ARCs assigned as plasmids or flanked by ICEs as potential conjugative mobility. Because plasmids and ICEs were the two major elements capable for conjugation transfer of ARG, while other MGEs like insertion sequences (IS), transposons and integrons rely on them for conjugation [13]. Furthermore, ARCs were taxonomically classified by fast approximation of evolutionary neighborhoods and a voting approach of all classified ORFs through the Taxator-tk (v1.3.3) and CAT, respectively [14,15].

## Viral contigs identification, clustering and taxonomic assignment

Viral contigs were identified, clustered and taxonomically assigned following our previous study [16]. Briefly, DNA contigs longer than 5.0 kb were collected, de-replicated, and screened using VirSorter2, VirFinder and geNomad [17,18]. The resulting viral contigs were merged and further de-replicated with CD-HIT (v4.7). A total of 13,124 high-confidence viral contigs were clustered into viral operational taxonomic units (vOTUs) at the species level using ClusterGenomes, with thresholds of 95% average nucleotide identity (ANI) and 85% alignment fraction (AF) [19]. This resulted in 6210 vOTUs.

ONT long reads from the composite stage were used to improve vOTU quality. Long reads were individually assembled into contigs using metaFlye v2.9-b1768.32. Viral contigs from long-read assemblies were identified, merged, and de-replicated as described above. Quickmerge was then used to refine the 6,210 vOTUs, with the original vOTU set as the reference.

Taxonomic assignment of vOTUs was performed using five complementary approaches: vConTACT2 [20], a majority-rule approach [21,22], BLASTn searches against the IMG/VR v3.0 and RefSeq viral databases, CAT, and geNomad.

Viral lifestyle was predicted by integrating the results from CheckV, VIBRANT, and PhaTYP. CheckV identified temperate viruses based on proviral integration sites or integrase genes [23]. VIBRANT v1.2.1 used a hybrid machine-learning and protein-similarity approach [24], while PhaTYP predicted lifestyle based on characteristic protein composition and association patterns [25]. A vOTU was classified as temperate if it met at least one of the following criteria: identified as a provirus by CheckV, labeled as “lysogenic” by VIBRANT, or assigned a temperate score ≥0.8 by PhaTYP. vOTUs that did not meet any of these criteria were considered potentially lytic or virulent [26].

To assess the novelty of the recovered vOTUs, predicted viral ORFs were compared against the IMG/VR v3.0 and RefSeq viral protein databases using DIAMOND with the parameters -id 30, -query-cover 50, and -evalue 1e-5.

**Identification of RNA vOTUs along with ARGs and host linkage analysis**

We also identified the RNA viruses based on the meta-transcriptomics through the RdRp homology and geNomade (gene marker and machine learning) [27,28]. RNA viral contigs were first identified based on homology searches using profile hidden Markov model approaches (HMMs) of RdRp domains (doi:10.5281/zenodo.5731488). To increase detection of divergent RdRp domain sequences, profile HMMs were generated and updated over ten iterations by recruiting newly detected sequences from our study as described previously [27]. The portion aligning to the HMM was trimmed by using HMMsearch (HMMER 3.1) with the flag -A for the hits with a bit score ≥30. Only sequences longer than 70% of the average length of the best-matching profile HMM were recruited and clustered to generate new HMMs by using the vFam pipeline with default parameters. The original HMM length was kept after each iteration to calculate the length fraction of the footprint. Then, the RNA viral contigs was identified through the geNomad with the end-to-end command under the default parameters. Only the viral contigs assigned as the Riboviria were remained as the RNA viruses excluding the identified DNA viruses which was expressed revealed by meta-transcriptome. Then, the identified RNA viral contigs by RdRp homology and geNomad were combined and redundancy removed through cd-hit-est (-c 1.0). Finally, the valid 1435 viral contigs were subjected to species-level clustering to create vOTUs using the ClusterGenomes scripts, following the MIUViG recommended criteria of 90% average nucleotide identity (ANI) and 80% alignment fraction (AF), resulting in the identification of 517 RNA vOTUs.

The quality of the RNA vOTUs was checked through checkV, and the lifestyle was also predicated. The ORFs were predicted using Prodigal v2.6.3 (-meta), and then searched against the database of CARD (–query-cover 70, –id 80, –e-value 1e-10) for the identification of ARGs. Taxonomic assignment of these vOTUs was carried out using three methods: 1) vConTACT2 [20]; 2) CAT; and 3) geNomad. We also tried to establish the RNA virus-host linkage based on CRISPR-Cas spacer, tRNA, homology match method and RdRp protein sequence similarity to endogenous virus elements (EVEs) between RNA vOTUs and MAGs, although none host linkage between these RNA phages and MAGs was identified.

## Profiling antiviral defense systems at MAGs level

Metagenome assembly genomes (MAGs) based on the short- and long-read metagenomics were collected as follows: 1) by binning using metaBAT2, MaxBin2 and CONCOCT, respectively; 2) by refining through Bin_refinement module in MetaWRAP (v1.3.0) with completeness＞80% and contamination＜5%; 3) and by combining and de-replicating through dRep [29]. To improve MAG quality, we identified and removed contaminating contigs from each MAG using MAGpurify v2.1.2 with the default modules and parameters [30]. The average completeness and contamination of the final collected MAGs is 91.6% and 1.6%, respectively, which indicated the high quality of these MAGs according to Minimum Information for a metagenome-assemble genome (MIMAG) standards [31]. MAGs were taxonomically classified by GTDB-Tk (v1.5.0, R214) [32], and considered as putative ARB if they carry ARGs [33].

ADS were detected using PADLOC (version 1.1.0) and DefenseFinder (version 1.0.8.22) as previously described [34,35]. ADS outputted by PADLOC in categories “other and adaptation” were removed, while ADS predicted by DefenseFinder to be located on different contigs or longer than 30kb were discarded. Then, the ADS were combined and de-replicated as the final. When integrating the outputs, DefenseFinder results were prioritized to resolve duplicate or conflicting predictions. Such conflicts were rare and primarily consisted of minor nomenclature variations rather than functional contradictions, and prioritizing DefenseFinder ensured a standardized classification across the dataset.

## In-memory construction of virus-host interactions

In-memory virus-host linkage was recalled through CRISPR-Cas spacers, tRNA and homology match method between viral contigs and binned MAGs as described in our previous study [16]. Briefly, vOTUs were searched against the curated CRISPR-Cas spacer database from MAGs using blastn-short with 100% identity and 100% coverage [36]; The tRNA sequences from vOTUs were blasted against MAGs with the parameters of 100% identity and 100% coverage without self-hits and duplicates. The best hits below an e-value threshold of 10^−5^ were considered as a match when phages aligned with more than 80% sequence identity over a length between 1 kb and 50% of the microbial host contig [37]. The CRISPR-Cas spacer–to-protospacer matches can also reveal the host adaptive immunity against specific viruses, plasmids or DNA sequences, which was used to build the immunity network if vOTUs or ARCs carried the protospacer same to the spacers of MAGs. The abundance and expression of target sequences including CRISPR-Cas spacer, genes, contigs and MAGs were calculated as RPKM through coverM.

## Hi-C construction of on-going virus-host infections

The five Hi-C libraries covering Stage II to VI were prepared from the composite stage samples using the ProxiMeta™ Hi-C preparation kit (Phase Genomics, Seattle, WA, USA). Hi-C libraries were generated using restriction enzymes Sau3AI and sequenced through the HiSeq 4000 platform (paired-end, 150 bp) with 30 Gb per sample. Hi-C reads were quality-controlled via the same method that was applied to the short-read metagenomics, and then mapped against a combined contigs database of vOTUs and MAGs using BWA mem (v0.7.17) with flag -5SP options specified to reduce the secondary and alternative mappings. Samtools (v1.17, view -F 0x904) is applied to remove unmapped reads, supplementary alignments, and secondary alignments, and then the BAM file is sorted by name using samtools sort. MetaCC is adopted to eliminate the systematic biases of Hi-C contacts through the module NormCC [38]. The filtered Hi-C contacts were then used to generate the on-going virus-host interaction network, where at least >5 Hi–C reads linkage exists for each pair, and the linked phage and host are at least simultaneously present (base coverage >70%) in one sample at that stage.

## Isolation and validation of antiviral defense strains and conjugation assays

*Escherichia coli* K12 (carrying plasmid RP4 with resistance to tetracycline, kanamycin, and ampicillin) and *E. coli* HB101 (streptomycin-resistant), were selected as model ARG hosts based on environmental prevalence. Virulent phages K12P4 and HB101P2, capable of infecting *E. coli* K12 and *E. coli* HB101, respectively, were isolated from domestic wastewater using standard double-layer agar methods.

Phages (10⁷ PFU/mL) and host bacteria (10⁶ CFU/mL, multiplicity of infection [MOI] = 10) were mixed and plated on double-layer agar, incubated at 37°C for 24 hours (six replicates). Surviving colonies were counted at 24, 36, and 48 hours, and antiviral defense mutation frequency was calculated as the ratio of surviving colonies to initial inoculated bacteria. Three resistant colonies per strain (e.g., K12-M1–M3, HB101-M1–M3) were selected for validation. Phage sensitivity was assessed via spot tests: 50 μL of overnight-cultured resistant strains (10⁸ CFU/mL) was spotted on agar, dried, and overlaid with 5 μL of phage (10⁸ PFU/mL). Plates were incubated at 37°C for 12 hours, and bacterial spot clarity was quantified using ImageJ. Additionally, resistant strains were co-cultured with phages at optimal MOI (K12P4: 0.001, HB101P2: 0.01) for 5 hours to monitor phage titers and bacterial growth, with non-resistant strains as controls.

Donor (*E. coli* K12) and recipient (*E. coli* HB101) bacteria (OD600 = 0.5, 800 μL each) were mixed in 2 mL EP tubes and incubated anaerobically at 30°C for 24 hours in triplicate. Conjugants, donors, and recipients were quantified on selective antibiotic plates. Conjugation frequency was calculated as the ratio of conjugants to recipients. A total of 16 conjugation assays, including controls, were performed in triplicate.

## Statistical Analysis

All statistical analyses and data visualizations were conducted using R (version 4.2.2) and Python (version 3.9), with a predetermined two-sided alpha level of 0.05 considered statistically significant. Missing data in the omics count matrices were treated as true zeros, and samples lacking operational metadata were excluded pairwise. To strictly control for Type I errors in high-dimensional omics screens, the Benjamini-Hochberg false discovery rate (FDR) procedure was systematically applied to report adjusted *p*-values. Non-parametric methods were employed, using the Kruskal-Wallis test followed by Fisher's Least Significant Difference (LSD) post-hoc test on ranks for multiple comparisons and the two-sided Wilcoxon rank-sum test for two-group comparisons. Bivariate associations were analyzed using Spearman's rank-order correlation to account for non-linear monotonic ecological data.

# SUPPLEMENTARY FIGURES


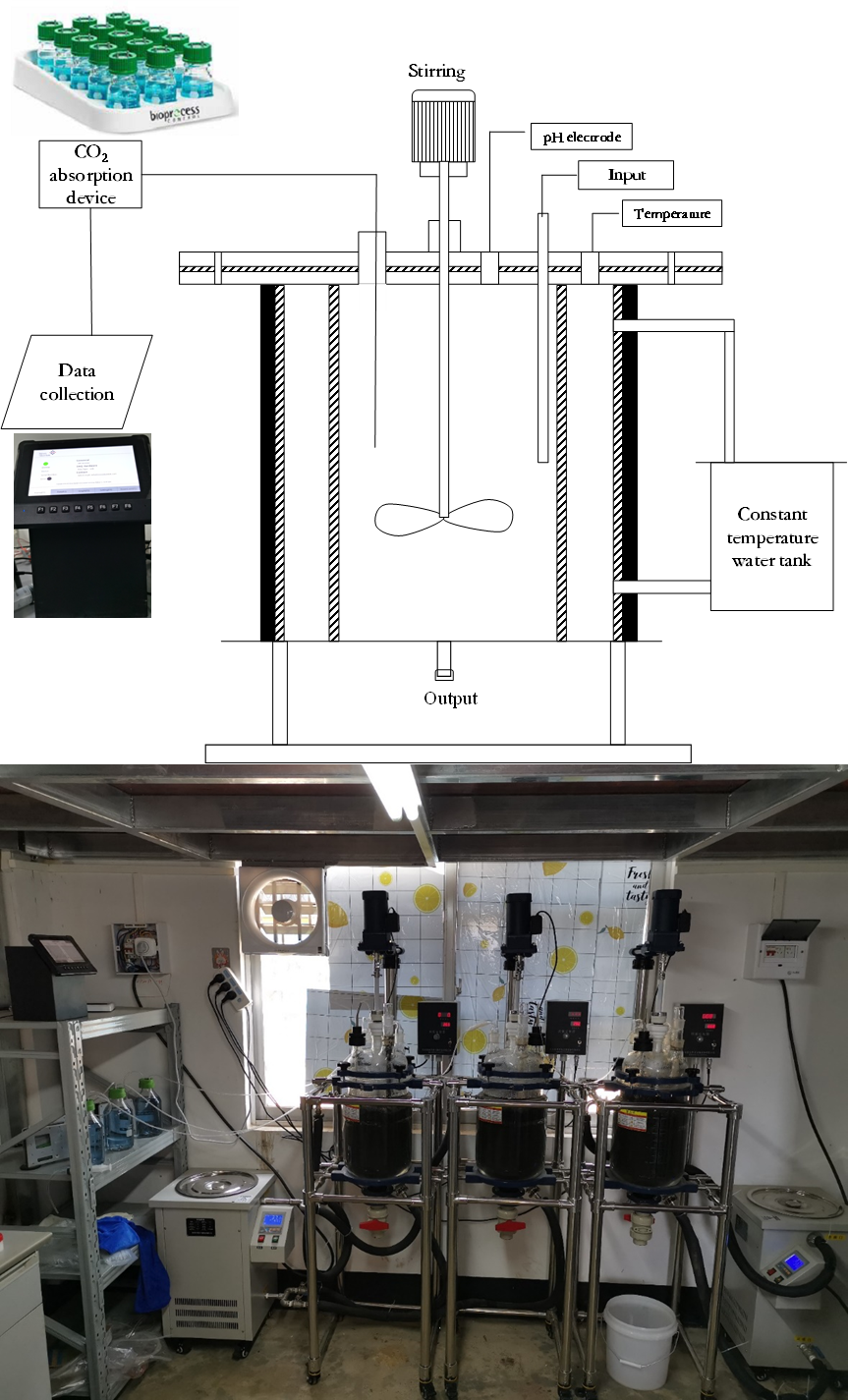


**Figure S1**. The schematic and in-site demonstration of the three continuous stirred tank reactors (CSTR) used in this study.

**Figure S2.** **Profile of antimicrobial resistance genes (ARGs) in anaerobic digestion.** Heatmap showing the dynamical changes of the top 10 ARGs in each sample (A), and the value is log2 transformed; (B) Principal component analysis (PCoA) showing the dynamics of ARGs profile along with varied operational parameters; Changes of selected ARGs and 16s rRNA determined through qPCR (C).

**Figure S3.** **Profile of the expression of antimicrobial resistance genes (ARGs) in anaerobic digestion.** Expression of ARGs response to the dynamics of operational parameters according to antibiotic class (A) and resistance mechanisms (B), while heatmap (C) showing the expression of ARGs subtypes in anaerobic digestion (log2 transformed of the RPKM value).

**Figure S4.** **Profile of the hosts of antimicrobial resistance genes (ARGs) and phages.** Overview of ARGs hosts at RNA level (A); Overall pattern and changes of the hosts infected by phages reflected by Hi-C (B).

**References**

1. Zhang, Junya, Tiedong Lu, Yufeng Chai, Qianwen Sui, Peihong Shen, Yuansong Wei. 2019. “Which animal type contributes the most to the emission of antibiotic resistance genes in large-scale swine farms in China?” *Science of the Total Environment* 658: 152–159. https://doi.org/10.1016/j.scitotenv.2018.12.175

2. Zhu, Yong-Guan, Timothy A. Johnson, Jian-Qiang Su, Min Qiao, Guang-Xia Guo, Robert D. Stedtfeld, et al. 2013. “Diverse and abundant antibiotic resistance genes in Chinese swine farms.” *Proceedings of the National Academy of Sciences of the United States of America* 110: 3435–3440. https://doi.org/10.1073/pnas.1222743110

3. Zhang, Junya, Tiedong Lu, Yuan Xin, Yuansong Wei. 2022. “Ferric Chloride Further Simplified the Horizontal Gene Transfer Network of Antibiotic Resistance Genes in Anaerobic Digestion.” *Science of the Total Environment* 844: 157054. https://doi.org/10.1016/j.scitotenv.2022.157054

4. Lu, Tiedong, Junya Zhang, Yuansong Wei, Peihong Shen. 2019. “Effects of ferric oxide on the microbial community and functioning during anaerobic digestion of swine manure.” *Bioresource Technology* 287: 121393. https://doi.org/10.1016/j.biortech.2019.121393

5. Uritskiy, Gherman V, Jocelyne DiRuggiero, James Taylor. 2018. “MetaWRAP - A flexible pipeline for genome-resolved metagenomic data analysis.” *Microbiome* 6: 158. https://doi.org/doi.org/10.1186/s40168-018-0541-1

6. Buchfink, Benjamin, Klaus Reuter, Hajk-Georg Drost. 2021. “Sensitive protein alignments at tree-of-life scale using DIAMOND.” *Nature Methods* 18: 366–368. https://doi.org/10.1038/s41592-021-01101-x

7. Alcock, Brian P, Amogelang R. Raphenya, Tammy T. Y. Lau, Kara K. Tsang, Mégane Bouchard, Arman Edalatmand, et al. 2020. “CARD 2020: Antibiotic resistome surveillance with the comprehensive antibiotic resistance database.” *Nucleic Acids Research* 48: D517–D525. https://doi.org/10.1093/nar/gkz935

8. Zhang, Junya, Qianwen Sui, Juan Tong, Chulu Buhe, Rui Wang, Meixue Chen, et al. 2016. “Sludge Bio-drying: Effective to Reduce both Antibiotic Resistance Genes and Mobile Genetic Elements.” *Water Research* 106: 62–70. http://dx.doi.org/10.1016/j.watres.2016.09.055

9. Zhang, Junya, Meixue Chen, Qianwen Sui, Juan Tong, Chao Jiang, Xueting Lu, et al. 2016. “Impacts of addition of natural zeolite or a nitrification inhibitor on antibiotic resistance genes during sludge composting.” *Water Research* 91: 339–349. https://doi.org/10.1016/j.watres.2016.01.010

10. Li, Dinghua, Ruibang Luo, Chi-Man Liu, Chi-Ming Leung, Hing-Fung Ting, Kunihiko Sadakane, et al. 2016. “MEGAHIT v1.0: A fast and scalable metagenome assembler driven by advanced methodologies and community practices.” *Methods* 102: 3–11. https://doi.org/10.1016/j.ymeth.2016.02.020

11. Bi, Dexi, Zhen Xu, Ewan M. Harrison, Cui Tai, Yiqing Wei, Xinyi He, et al. 2012. “ICEberg: A web-based resource for integrative and conjugative elements found in Bacteria.” *Nucleic Acids Research* 40: 621–626. https://doi.org/10.1093/nar/gkr846

12. Krawczyk, Pawel S, Leszek Lipinski, Andrzej Dziembowski. 2018. “PlasFlow: predicting plasmid sequences in metagenomic data using genome signatures.” *Nucleic Acids Research* 46: e35. https://doi.org/10.1093/nar/gkx1321

13. Khedkar, Supriya, Georgy Smyshlyaev, Ivica Letunic, Oleksandr M. Maistrenko, Luis Pedro Coelho, Askarbek Orakov, et al. 2022. “Landscape of mobile genetic elements and their antibiotic resistance cargo in prokaryotic genomes.” *Nucleic Acids Research* 50: 3155–3168. https://doi.org/10.1093/nar/gkac163

14. Dröge, J, I. Gregor, A. C. McHardy. 2015. “Taxator-tk: Precise taxonomic assignment of metagenomes by fast approximation of evolutionary neighborhoods.” *Bioinformatics* 31: 817–824. https://doi.org/10.1093/bioinformatics/btu745

15. Bastiaan von Meijenfeldt, F. A, Ksenia Arkhipova, Diego D. Cambuy, Felipe H. Coutinho, Bas E. Dutilh. 2019. “Robust taxonomic classification of uncharted microbial sequences and bins with CAT and BAT.” *Genome Biology* 20: 217. https://doi.org/10.1186/s13059-019-1817-x

16. Zhang, Junya, Tiedong Lu, Yunpeng Song, Ulisses Nunes da Rocha, Jibao Liu, Marcell Nikolausz, et al. 2024. “Viral Communities Contribute More to the Lysis of Antibiotic-Resistant Bacteria than the Transduction of Antibiotic Resistance Genes in Anaerobic Digestion Revealed by Metagenomics.” *Environmental Science & Technology* 58: 2346–2359. https://doi.org/10.1021/acs.est.3c07664

17. Guo, Jiarong, Ben Bolduc, Ahmed A. Zayed, Arvind Varsani, Guillermo Dominguez-Huerta, Tom O. Delmont, et al. 2021. “VirSorter2: a multi-classifier, expert-guided approach to detect diverse DNA and RNA viruses.” *Microbiome* 9: 37. https://doi.org/10.1186/s40168-020-00990-y

18. Ren, Jie, Nathan A. Ahlgren, Yang Young Lu, Jed A. Fuhrman, Fengzhu Sun. 2017. “VirFinder: a novel k-mer based tool for identifying viral sequences from assembled metagenomic data.” *Microbiome* 5: 69. https://doi.org/10.1186/s40168-017-0283-5

19. Roux, Simon, Evelien M Adriaenssens, Bas E Dutilh, Eugene V Koonin, Andrew M Kropinski, Mart Krupovic, et al. 2019. “Minimum information about an uncultivated virus genome (MIUVIG).” *Nature Biotechnology* 37: 29–37. https://doi.org/10.1038/nbt.4306

20. Jang, Ho Bin, Benjamin Bolduc, Olivier Zablocki, Jens H. Kuhn, Simon Roux, Evelien M. Adriaenssens, et al. 2019. “Taxonomic assignment of uncultivated prokaryotic virus genomes is enabled by gene-sharing networks.” *Nature Biotechnology* 37: 632–639. https://doi.org/10.1038/s41587-019-0100-8

21. Zheng, Xiaoxuan, Martin T. Jahn, Mingming Sun, Ville-Petri Friman, Jose Luis Balcazar, Jinfeng Wang, et al. 2022. “Organochlorine contamination enriches virus-encoded metabolism and pesticide degradation associated auxiliary genes in soil microbiomes.” *The ISME Journal* 16: 1397–1408. https://doi.org/10.1038/s41396-022-01188-w

22. Gregory, Ann C, Olivier Zablocki, Ahmed A. Zayed, Allison Howell, Benjamin Bolduc, Matthew B. Sullivan. 2020. “The Gut Virome Database Reveals Age-Dependent Patterns of Virome Diversity in the Human Gut.” *Cell Host & Microbe* 28: 724–740. https://doi.org/10.1016/j.chom.2020.08.003

23. Stephen Nayfach, Antonio Pedro Camargo, Frederik Schulz, Emiley Eloe-Fadrosh, Simon Roux, Nikos C. Kyrpides. 2021. “CheckV assesses the quality and completeness of metagenome-assembled viral genomes.” *Nature Biotechnology* 39: 578–585. https://doi.org/10.1038/s41587-020-00774-7

24. Kieft, Kristopher, Zhichao Zhou, Karthik Anantharaman. 2020. “VIBRANT: Automated recovery, annotation and curation of microbial viruses, and evaluation of viral community function from genomic sequences.” *Microbiome* 8: 90. https://doi.org/10.1186/s40168-020-00867-0

25. Shang, Jiayu, Cheng Peng, Herui Liao, Xubo Tang, Yanni Sun. 2023. “PhaBOX: A web server for identifying and characterizing phage contigs in metagenomic data.” *Bioinformatics Advances* 3: vbad101. https://doi.org/10.1093/bioadv/vbad101

26. Wang, Tao, Peiyu Zhang, Karthik Anantharaman, Huan Zhang, Min Zhang, et al. 2025. “Metagenomic analysis reveals how multiple stressors disrupt virus–host interactions in multi-trophic freshwater mesocosms.” *Nature Communications* 16: 7806. https://doi.org/10.1038/s41467-025-63162-2

27. Zayed, Ahmed A, James M. Wainaina, Guillermo Dominguez-Huerta, Eric Pelletier, Jiarong Guo, Mohamed Mohssen, et al. 2022. “Cryptic and abundant marine viruses at the evolutionary origins of Earth’s RNA virome.” *Science* 376: 156–162. https://doi.org/10.1126/science.abm5847

28. Camargo, Antonio Pedro, Michal Babinski, Yan Xu, Simon Roux, Frederik Schulz, Bin Hu, et al. 2023. “Identification of mobile genetic elements with geNomad.” *Nature Biotechnology* 42: 1303–1312. https://doi.org/10.1038/s41587-023-01953-y

29. Olm, Matthew R, Christopher T Brown, Brandon Brooks, Jillian F Banfield. 2017. “dRep: A tool for fast and accurate genomic comparisons that enables improved genome recovery from metagenomes through de-replication.” *The* *ISME Journal* 11: 2864–2868. https://doi.org/10.1038/ismej.2017.126

30. Nayfach, Stephen, Zhou Jason Shi, rekha Seshadri, Katherine S. Pollard, Nikos c. Kyrpides. 2019. “New insights from uncultivated genomes of the global human gut microbiome.” *Nature* 568: 505–510. https://doi.org/10.1038/s41586-019-1058-x

31. Bowers, Robert M, Nikos C Kyrpides, Ramunas Stepanauskas, Miranda Harmon-Smith, Devin Doud, T B K Reddy, et al. 2017. “Minimum information about a single amplified genome (MISAG) and a metagenome-assembled genome (MIMAG) of bacteria and archaea.” *Nature Biotechnology* 35: 725–731. https://doi.org/10.1038/nbt.3893

32. Chaumeil, Pierre-Alain, Aaron J. Mussig, Philip Hugenholtz, Donovan H. Parks. 2020. “GTDB-Tk: A toolkit to classify genomes with the genome taxonomy database.” *Bioinformatics* 36: 1925–1927. https://doi.org/10.1093/bioinformatics/btz848

33. Sayers, Samantha, Edison Ong, Shunzhou Deng, Guanghua Fu,YuLin, et al. 2018. “Victors: a web-based knowledge base of virulence factors in human and animal pathogens.” *Nucleic Acids Research* 47: D693–D700. https://doi.org/10.1093/nar/gky999

34. Payne, Leighton J, Thomas C. Todeschini, YiWu, Benjamin J. Perry, Clive W. Ronson, Peter C. Fineran, et al. 2021. “Identification and classification of antiviral defence systems in bacteria and archaea with PADLOC reveals new system types.” *Nucleic Acids Research* 49: 10868–10878. https://doi.org/10.1093/nar/gkab883

35. Tesson, Florian, Alexandre Hervé, Ernest Mordret, Marie Touchon, Camille d’Humières, Jean Cury, et al. 2022. “Systematic and quantitative view of the antiviral arsenal of prokaryotes.” *Nature Communications* 13: 2561. https://doi.org/10.1038/s41467-022-30269-9

36. Wu, Ruonan, Michelle R. Davison, William C. Nelson, Montana L. Smith, Mary S. Lipton, Janet K. Jansson, et al. 2023. “Hi-C metagenome sequencing reveals soil phage–host interactions.” *Nature Communications* 14: 7666. https://doi.org/10.1038/s41467-023-42967-z

37. Jahn, M. T, T. Lachnit, S. M. Markert, C. Stigloher, L. Pita, M. Ribes, et al. 2021. “Lifestyle of sponge symbiont phages by host prediction and correlative microscopy.” *The ISME Journal* 15: 2001–2011. https://doi.org/10.1038/s41396-021-00900-6

38. Du, Yuxuan, Fengzhu Sun. 2023. “MetaCC allows scalable and integrative analyses of both long-read and short-read metagenomic Hi-C data.” *Nature Communications* 14: 6231. https://doi.org/10.1038/s41467-023-41209-6
